# Beyond the Scavenging of Reactive Oxygen Species (ROS): Direct Effect of Cerium Oxide Nanoparticles in Reducing Fatty Acids Content in an In Vitro Model of Hepatocellular Steatosis

**DOI:** 10.3390/biom9090425

**Published:** 2019-08-29

**Authors:** Marina Parra-Robert, Eudald Casals, Nuria Massana, Muling Zeng, Meritxell Perramón, Guillermo Fernández-Varo, Manuel Morales-Ruiz, Víctor Puntes, Wladimiro Jiménez, Gregori Casals

**Affiliations:** 1 Service of Biochemistry and Molecular Genetics, Hospital Clinic Universitari, Centro de Investigación Biomédica en Red de Enfermedades Hepáticas y Digestivas (CIBERehd), Institut d’Investigacions Biomèdiques August Pi i Sunyer (IDIBAPS), 08905 Barcelona, Spain; 2 School of Biotechnology and Health Sciences, Wuyi University, Jiangmen 529020, China; 3 Departament of Biomedicine, University of Barcelona, 08905 Barcelona, Spain; 4 Working group for the biochemical assessment of hepatic disease-SEQC^ML^, 08036 Barcelona, Spain; 5 Vall d’Hebron Research Institute (VHIR), 08035 Barcelona, Spain; 6 Institut Català de Nanociència i Nanotecnologia (ICN2), CSIC, The Barcelona Institute of Science and Technology (BIST), Campus UAB, Bellaterra, 08193 Barcelona, Spain; 7 Institució Catalana de Recerca i Estudis Avançats (ICREA), 08010 Barcelona, Spain

**Keywords:** nonalcoholic fatty liver disease, steatosis, liver, cerium oxide nanoparticles, oxidative stress

## Abstract

Nonalcoholic fatty liver disease (NAFLD) is characterized by hepatic accumulation of lipids. Antisteatotic effects of cerium oxide nanoparticles (CeO_2_NPs) have recently been shown in animal models of liver disease. However, it is unclear whether the activity of CeO_2_NPs is related solely to the decrease in oxidative stress or, in addition, they directly decrease liver fatty acid accumulation. To address this question, in this work, we used an in vitro model of hepatocellular steatosis, exposing HepG2 cells to oleic and palmitic acid. Cell uptake of CeO_2_NPs and their effect on oxidative stress and viability of hepatic cells cultured with H_2_O_2_ were also evaluated. Results show that CeO_2_NPs were uptaken by HepG2 cells and reduced oxidative stress and improved cell viability. Treatment with oleic and palmitic acid increased lipogenesis and the content of different fatty acids. CeO_2_NPs reduced palmitic and stearic acid and most fatty acids consisting of more than 18 carbon atoms. These effects were associated with significant changes in elongase and desaturase activity. In conclusion, CeO_2_NPs directly protected HepG2 cells from cell injury in oxidative stress conditions and reduced fatty acid content in steatotic conditions by inducing specific changes in fatty acid metabolism, thus showing potential in the treatment of NAFLD.

## 1. Introduction

Nonalcoholic fatty liver disease (NAFLD) is characterized by hepatic lipid accumulation (steatosis), mainly in the form of triglycerides, and covers a wide spectrum of liver disease that ranges from simple hepatic steatosis (nonalcoholic fatty liver, NAFL) to nonalcoholic steatohepatitis (NASH), the last being characterized by the appearance of inflammation and a higher risk of progression to more advanced forms including fibrosis, cirrhosis and hepatocellular carcinoma (HCC) [1].

Oxidative stress plays a role in the molecular mechanisms behind the progression of NAFLD. In this regard, the pathogenesis of NAFLD may be explained by a multiple-hit hypothesis [2]. In the context of excess energy, fatty acids (FAs) are esterified into triacylglycerides and stored in lipid droplets in the liver. The multiple-hit hypothesis considers multiple insults, including insulin resistance, adipokines, nutritional factors or gut microbiota that act together on genetically predisposed subjects in the development and progression of the disease [2,3]. These insults are associated with increased oxidative stress, proinflammatory cytokines and adipocytokines, leading to hepatocyte injury, inflammation and fibrosis [4,5]. The hepatic accumulation of free FA, lipid peroxidation products (reactive aldehydes and oxysterols) and reactive oxygen species (ROS) contribute to the development and progression of NAFLD from simple steatosis to NASH by impairing mitochondrial function, energy balance and biogenesis adaptation to chronic injury [6,7].

The current treatment of NAFLD consists of diet and lifestyle interventions, such as healthy eating habits and physical activity, to achieve weight loss and the control of its associated cardio-metabolic risk factors [8,9,10]. However, reinforcement of these interventions by pharmacological therapies would be useful in the management of patients. Unfortunately, effective treatment options for NAFLD remain limited [1]. At present, there are no pharmacological treatments licensed for the reduction of liver steatosis, and the American and European guidelines for the management of NAFLD patients are only supportive of the use of two drugs: pioglitazone in patients with NASH with or without *diabetes mellitus*, and vitamin E, a recognized antioxidant, in nondiabetic patients with NASH [9,10]. The identification of strategies directed to halt NAFLD progression are, therefore, an urgent need.

In recent years, the antioxidant properties of cerium oxide nanoparticles (CeO_2_NPs) have been widely described in the literature [11,12,13,14,15,16]. CeO_2_NPs, which display minimal toxicity to normal tissues and provide cellular protection from various forms of ROS and irradiation [17], have emerged as a powerful antioxidant therapeutic tool in the prevention and treatment of oxidative-stress-related diseases. The interest in CeO_2_NPs include their natural advantages over other antioxidants since CeO_2_NPs participate catalytically in redox cycles, which mean that they are not consumed during reaction, and thus, they can be used at low doses. Their oxygen storage capacity has proven useful in scavenging free radicals as soon as they are generated in situations of metabolic imbalance [12]. In this context, it has been shown that CeO_2_NPs exhibit superoxide dismutase [18], catalase [19] and peroxidase [20] mimetic activities. Thus, CeO_2_NPs have been postulated as a possible treatment for pathologies associated with chronic oxidative stress and inflammation such as cancer, cardiac disease and neurodegenerative disease, among others [21,22,23,24,25]. Interestingly, after their administration, CeO_2_NPs are accumulated mainly in the liver and for long periods of time [26,27]. Recent studies carried out in our laboratory show that CeO_2_NPs decrease portal hypertension, liver inflammatory response and hepatic steatosis in a rat model of liver fibrosis induced by CCl_4_ [27]. Additionally, oral administration of CeO_2_NPs to a NAFLD rat model associated with neonatal monosodium-glutamate-induced obesity has been reported to decrease body weight and liver steatosis [28]. However, it is unknown whether this antisteatotic effect is due to a direct effect of CeO_2_NPs in the liver. In the current study, we used an in vitro model system of cultured human hepatic cells (HepG2) to test the hypothesis of whether CeO_2_NPs can directly reduce FA accumulation in the absence of other stimuli.

## 2. Materials and Methods

### 2.1. Synthesis and Characterization of CeO_2_NPs

#### 2.1.1. Synthesis

CeO_2_NPs of 4 nm of mean diameter were synthesized in aqueous media by the chemical precipitation of Cerium (III) Nitrate Hexahydrate (Ce(NO_3_)_3_) under basic conditions. Briefly, 10 mM of Cerium (III) Nitrate Hexahydrate was dissolved in 97 mL of Milli-Q H2O at room temperature. The solution was left under stirring for about 30 min. To this, 3 mL of tetramethylammonium hydroxide (TMAOH) (1.0 ± 0.02 M in H_2_O) was added under stirring (final TMAOH concentration of 30 mM), and the mixture was left under stirring overnight. Afterward, nanoparticles (NPs) were purified by centrifugation (20,000 g, 10 min) and resuspended in an aqueous solution of 1 mM TMAOH. In this synthesis, the conversion of initial cerium precursor to CeO_2_NPs is nearly complete, as determined by UV-VIS spectroscopy and inductively coupled plasma mass spectrometry (ICP-MS).

#### 2.1.2. Albumin Conjugation

Electrostatically stabilized CeO_2_NPs have a strong tendency to aggregate at physiological pH, which can result in deleterious immune responses [29]. To avoid this, Human Serum Albumin (HSA) was conjugated to the CeO_2_NPs surface prior to their use. Albumin was added at 100-fold the amount needed to cover the surface of CeO_2_NPs to ensure complete albumin corona formation. 

#### 2.1.3. Characterization

The NPs’ size was determined using high-resolution transmission electron microscopy (HR-TEM) with a microscope JEOL JEM 2010F at 200 kV (Tokyo, Japan), and further image analysis using the Image J free software (National Institutes of Health, Bethesda, MD, USA). The surface charge of the NPs was characterized in a Z-sizer (Malvern, Worcestershire, UK). The crystal structure was analyzed by X-ray powder diffraction (XRD) (Xpert Pannalytical, MA, USA), and the light interaction by UV-VIS spectroscopy (Shimadzu, Kyoto, Japan).

### 2.2. Cell Culture

HepG2 cells were obtained from American Type Culture Collection (ATCC, Manassas, VA, USA). This immortalized human hepatocellular stable cell line can be repeatedly frozen, thawed and propagated. HepG2 cells were seeded into either 96-well culture plates for cell bioavailability analysis, 24-well culture plates for ROS measurements, six-well culture plates for steatosis induction and nanoparticles cell uptake evaluation or 75 cm^2^ flasks for measurements of oxysterols. After seeding, cells were grown to confluence in Dulbecco’s Modified Eagle Medium (DMEM), supplemented with 10% fetal bovine serum (FBS), 50 U/mL penicillin and 50 μg/mL streptomycin, in a humidified atmosphere of 5% CO_2_ at 37 °C. Thereafter, the cells were switched to serum-free DMEM for 16 h. For cell stimulation and treatment, old medium was removed and replaced with medium, medium containing 1.5 mM H_2_O_2_ (Sigma-Aldrich, St. Louis, MO, USA) or medium containing 1.5 mM H_2_O_2_ and CeO_2_NPs. Cells were incubated for the indicated time points and then harvested for biochemical or molecular assays. All experiments were repeated at least three times. 

### 2.3. Internalization of CeO_2_NPs in HepG2 Cells

The uptake of CeO_2_NPs by the cell cultures was assessed using bright-field and dark-field TEM images. HepG2 cells were seeded (1.5x10^6^ cells/well) in six-well plates and incubated with or without CeO_2_NPs (10 µg/mL) for 24 h. Thereafter, cells were fixed in a 0.1 M phosphate buffer containing 2.5% glutaraldehyde and 2% paraformaldehyde. Cells were embedded in Spur’s resin and thin sections (50–55 nm) were cut and placed on copper grids and then stained with uranyl acetate and lead citrate. After staining, the sections were examined using a low-electron-power microscope to increase contrast in a JEOL-1010 TEM (JEOL, Tokyo, Japan) operated at 80 kV and equipped with a BioScan camera (Gatan, CA, USA).

### 2.4. Cell Viability Analysis

Cell viability was assessed using the MTS (3-(4,5-dimethylthiazol-2-yl)- 5-(3-carboxymethoxyphenyl)-2-(4-sulfophenyl)-2H-tetrazolium) technique (CellTiter 96; Promega, Madison, WI, USA) according to the manufacturer’s instructions. In brief, cells were seeded (1 × 10^5^ cells/well) in 96-well plates and, after overnight starvation, cells were treated with medium, H_2_O_2_ (1.5 mM) or H_2_O_2_ and CeO_2_NPs (10 µg/mL) for 90 min at 37 °C. Then, cells were washed twice with Hank’s Balanced Salt Solution (HBSS) and CellTiter reagent was added to each well. After incubation for 3 h at 37 °C to allow cells to bioreduce MTS into formazan, the absorbance of formazan, measured at 490 nm (SpectraMax 340, Molecular Devices, Sunnyvale, CA), is directly proportional to the number of living cells in culture.

### 2.5. Reactive Oxygen Species Measurement

Fluorescence spectrophotometry was used to measure ROS, with 2’,7’-DCF-DA (2’,7’-dichlorofluorescein diacetate) as the probe (Sigma-Aldrich, St. Louis, MO, USA). DCF-DA readily diffuses through the membrane and is enzymatically hydrolyzed by intracellular esterases to the nonfluorescent DCFH (2’,7’-dichlorodihydrofluorescein), which can then be rapidly oxidized to fluorescent DCF in the presence of ROS. HepG2 cells were seeded in 24-well plates and, after overnight starvation, cells were incubated alone or with H_2_O_2_ (1.5 mM) in the presence of CeO_2_NPs (1 µg/mL) or medium for 90 min. Thereafter, cells were washed with HBSS and incubated with 10-μM DCF-DA in Hanks Balanced Salt Solution for 40 min at 37 °C in the dark. The supernatant was collected to measure the production of ROS, and the intensity of DCF fluorescence was immediately read in a fluorescence spectrophotometer (FLUOstar OPTIMA; BMG LABTECH, Ortenberg, Germany) at 485 nm for excitation and at 520 nm for emission. 

### 2.6. Oxysterols Measurements

Cells were seeded in 75-cm^2^ flasks and, after overnight starvation, incubated with medium, H_2_O_2_ (1.5 mM) or H_2_O_2_ and CeO_2_NPs (1.5 mM; 10 µg/mL) for 90 min. Afterward, cell pellets were obtained by trypsinization and the oxysterol content was analyzed. Cholesterol, cholesterol d7, 7β-hydroxycholesterol, 7-ketocholesterol, 25-hydroxycholesterol and 5α-cholestane were purchased from Steraloids (Newport, RI). Lipids were extracted from HepG2 cells with chloroform–methanol (2:1vol/vol) in the presence of butylated hydroxytoluene (0.005%) as an antioxidant. 5α-cholestane and cholesterol-d7 were added as internal standards. After centrifugation, the lower phase was evaporated under a nitrogen stream and extracts were silylated with N,O-bis-(trimethylsilyl)-trifluoroacetamide containing 1% trimethylchlorosilane (Sigma). Gas chromatography mass spectrometry (GC–MS) analyses were performed on a Shimadzu GCMS QP2010 Ultra instrument (Kyoto, Japan). Sterols were separated in a Sapines-5MS+ capillary column (30 m × 0.25 mm internal diameter × 0.25 µm film thickness) from Teknokroma with helium as a carrier gas at a constant velocity of 50 cm/s. The temperature program was programmed to begin at 50 °C and this temperature was maintained for 3 min. Then, it was elevated at a rate of 80 °C min^−1^ to 240 °C, before being increased at 2 °C min^−1^ to a temperature of 290 °C and finally, maintained for 2 min at 290 °C. The ion source and transfer line temperatures were set to 270 °C and 280 °C, respectively. Mass detector was operated in selected ion monitoring (SIM) mode and identification of the analytes was achieved by GC retention time comparison with reference standards. No oxidation of d7-cholesterol to deuterated oxysterols was observed during the analytical procedure.

### 2.7. Induction of Steatosis in HepG2 Cells with Oleic and Palmitic Acids

HepG2 cells were seeded (1.5 × 10^6^ cells/well) in six-well plates and grown to confluence as described above. After overnight starvation, the medium was changed to 10% FBS medium or 10% FBS medium with 1.33 mM oleic acid (OA) and 0.67 mM palmitic acid (PA) with or without CeO_2_NPs (10 µg/mL) for 24 h to induce the hepatosteatotic condition [30,31]. Three cell cultures were compared: (i) cells incubated with 10% FBS medium, (ii) cells incubated with 10% FBS medium and OA+PA, (iii) cells incubated with 10% FBS and OA+PA and CeO_2_NPs (10 µg/mL).

### 2.8. Total FA Measurements

Lipids were extracted from HepG2 cells with chloroform–methanol (2:1 vol/vol) in the presence of butylated hydroxytoluene (0.005%). C19:0 (Sigma Aldrich, Madrid, Spain) was used as an internal standard. After homogenization, samples were placed on an orbital shaker for 20 min at room temperature and centrifuged for 10 min at 10,000 rpm. The supernatant was mixed with 250 μL of water, vortexed and centrifuged again for 10 min at 10,000 rpm. The lower organic phase was retrieved, evaporated under a nitrogen stream and redissolved in a mixture of methanol:hexane (4:1, v/v). Acetyl chloride was added and FA methyl ester (FAME) obtained by heating the samples at 100 °C for 1 h. GC–MS analyses were performed on a Shimadzu GCMS QP2010 Ultra instrument (Kyoto, Japan). Final extracts were injected into the gas chromatograph interfaced with a mass selective detector. Chromatographic separation was achieved on a Sapines-5MS+ capillary column (30 m × 0.25-mm internal diameter × 0.25-μm film thickness) from Teknokroma (Barcelona, Spain) with helium as a carrier gas at a constant velocity of 50 cm/s. The temperature program was set to begin at 50 °C and this temperature was maintained for 3 min. Then, it was elevated at a rate of 80 °C min^−1^ to 240 °C, before being increased at 2 °C min^−1^ until 290 °C and finally, maintained for 2 min at 290 °C. The ion source and transfer line temperatures were set to 270 °C and 280 °C, respectively. Mass detector was operated in scan mode. Identification of the FAME in the sample extracts was achieved by mass spectrum and GC retention time comparison with reference standards. The activities of FA elongases and desaturases were estimated, assessing established product to precursor metabolic ratios for each reaction [32,33,34]. In addition, the lipogenic index was calculated as the ratio C16:0/C18:2n6c [35,36].

### 2.9. Statistical Analysis

Quantitative data were analyzed using GraphPad Prism 6 (GraphPad Software Inc., San Diego, CA, USA) and statistical analysis of the results was performed by an unpaired *t*-test. Data is expressed as mean ± standard error of the mean (SEM) and considered significant at a *p*-level of 0.05 or less. The study was performed according to the criteria of the Investigation and Ethics Committee of the Hospital Clínic of Barcelona.

## 3. Results

### 3.1. Characterization of CeO_2_NPs

**Figure 1:**
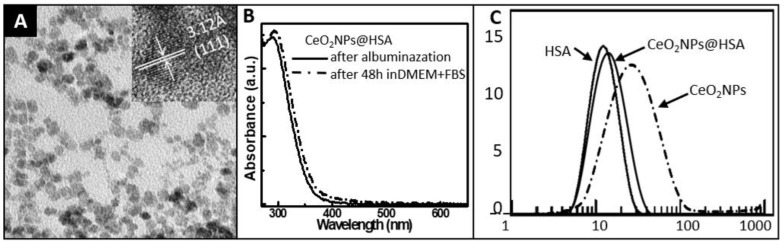
CeO_2_NPs characterization. (**a**) Transmission electron microscopy (TEM) and high-resolution transmission electron microscopy (HR-TEM) (inset) showing the as-synthesized cerium oxide (CeO_2_) nanocrystals of 4±1 nm; (**b**) UV-VIS spectroscopy of CeO_2_NPs after conjugation to Human Serum Albumin (HSA) (CeO_2_NPs@HSA) and the same nanoparticles (NPs) after 48 h in model physiological media (DMEM + Fetal Bovine Serum at 10% v/v). The similar spectra through time shows the stability in the physiological media of the NPs used; (**c**) Dynamic light scattering (DLS) of CeO_2_NPs as synthesized, HSA, and CeO_2_NPs@HSA. While the spectrum of CeO_2_NPs shows a large presence of agglomerated NPs, the similar spectra of HSA and CeO_2_NPs@HSA rules out the presence of CeO_2_ aggregates. Note, that all samples have been purified before measurement.

A description of the characterization of the CeO_2_NPs used in this study has been published previously [27]. Briefly, the measured Z-Potential of CeO_2_NPs was +40 mV at the final synthesis pH (pH = 6). The X-ray diffraction pattern showed pure CeO_2_NPs with the typical peak broadening characteristic of nanosized particles. In this synthesis, CeO_2_NPs obtained are monodisperse with an average of 4±1 nm size, as determined by TEM. In these conditions, the high surface energy of the NPs makes their electrical double layer unable to keep them apart and small agglomerates are formed. To avoid that, their immediate albuminization as they are formed, once the extreme initial pH of the solution is reduced (from pH 12 to pH 6), is key. HSA conjugation on the surface of the CeO_2_NPs has been characterized by the increase in DLS and modification of the Z-Potential (from +40 mV of NPs as synthesized to −14 mV, which is the average Z-Potential value of the HSA (Figure 1).

### 3.2. Cellular Uptake of CeO_2_NPs

**Figure 2:**
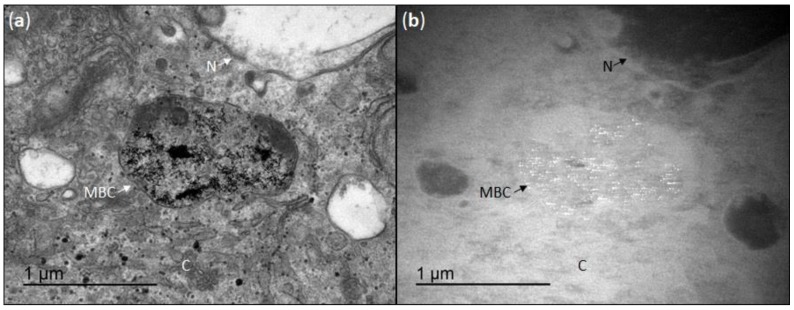
Cellular uptake of CeO_2_NPs. (**a**) Transmission electron microscopy image of CeO_2_NPs in HepG2 cells that were incubated for 24 h at a CeO_2_NPs concentration of 10 µg/mL; (**b**) the same image obtained under dark-field exposure, since the high contrast of the CeO_2_NPs allows them to be easily distinguished. MBC indicates a membrane-bound compartment, N the nucleus and C the cytoplasm.

Transmission electron microscopy was used to evaluate the association between HepG2 cells and incubation with CeO_2_NPs (10 µg/mL) for 24 h. TEM images revealed the presence of CeO_2_NPs inside the cells, mainly in membrane-bound compartments and to a lesser extent, free in the cytoplasm (Figure 2). The presence of CeO_2_NPs is revealed in the dark-field images since the high contrast of cerium oxide (CeO_2_) crystals allowed them to be easily distinguished (as bright spots) from other cellular structures. The confinement of CeO_2_NPs mainly in vacuoles or endosome-like organelles suggested their presence in secretory compartments. These structures were not observed in the TEM images obtained from controls cells incubated without CeO_2_NPs.

### 3.3. Effect of CeO_2_NPs in the Viability of HepG2 Incubated with H_2_O_2_

To evaluate cellular CeO_2_NPs protection from H_2_O_2_-induced cytotoxicity in human hepatic cells, HepG2 cells were incubated with H_2_O_2_ and cellular viability was analyzed in the presence of CeO_2_NPs or vehicle. Oxidative stress induced with a 1.5 mM H_2_O_2_ produced a reduction in cell viability of 14% (control 100% ± 1.8% and H_2_O_2_ 85.8% ± 1.1% viability (mean ± SEM); *p* < 0.0001). In these conditions, CeO_2_NPs treatment improved cell viability, from 85.8% ± 1.1% in the H_2_O_2_ condition to 99.6% ± 1.8% in cells exposed to H_2_O_2_ and treated with NPs (*p* < 0.0001), indicating that CeO_2_NPs protect from the oxidative-stress-associated cell death induced by H_2_O_2_ in HepG2 cells (Figure S1A).

### 3.4. Effect of CeO_2_NPs in the ROS Production of HepG2 Incubated with H_2_O_2_

HepG2 cells incubated with H_2_O_2_ (1.5 mM) increased ROS production by 25% (control 100% ± 3.9% and H_2_O_2_ 125.4% ± 3.3% (mean ± SEM); *p* < 0.0001), as determined by fluorescence spectrophotometry using the oxidant-sensitive dye 2´,7´-DCF-DA. ROS production was significantly inhibited in the presence of CeO_2_NPs (H_2_O_2_ 125.4% ± 3.3% and H_2_O_2_ with CeO_2_NPs 109.8% ± 3.8% (mean ± SEM); *p* = 0.006). These results indicate that CeO_2_NPs protect from the oxidative stress induced by H_2_O_2_ in HepG2 cells (Figure S1B).

### 3.5. Effect of CeO_2_NPs in Cholesterol and Oxysterols Content of HepG2 Cells Treated with H_2_O_2_

**Figure 3:**
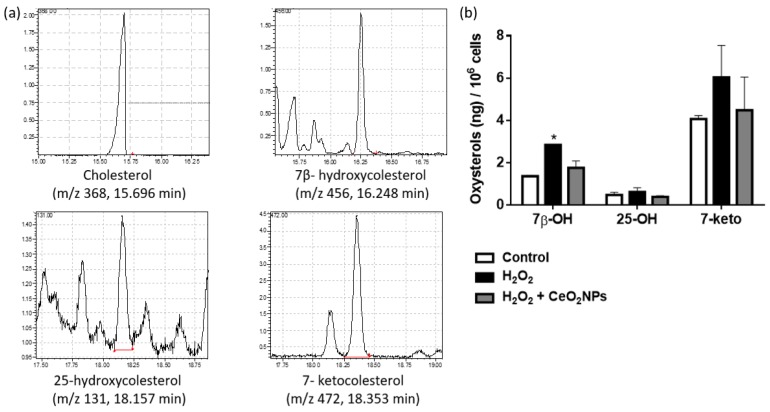
(**a**) Gas chromatography mass spectrometry chromatograms of cholesterol and its oxidation products in HepG2 cells; (**b**) oxysterols content in HepG2 cells treated with vehicle, H_2_O_2_ (1.5 mM) and H_2_O_2_ and CeO_2_NPs (1.5 mM; 10 µg/mL). * *p* < 0.05 compared with control.

Effects of CeO_2_NPs in the cellular content of cholesterol and cholesterol oxidation products were evaluated in HepG2 cells treated with vehicle, HepG2 cells treated with H_2_O_2_ (1.5 mM) and HepG2 cells treated with H_2_O_2_ and CeO_2_NPs (1.5 mM, 10 µg/mL). Figure 3A shows a chromatogram of cholesterol and cholesterol oxidation products obtained by GC–MS in HepG2 cells. Cholesterol content was approximately 1000-fold higher than cholesterol oxidation products. The most abundant oxysterol measured was 7-ketocholesterol, followed by 7β-hydroxycholesterol and 25-hydroxycholesterol. No significant differences in cholesterol content were observed between cells treated with vehicle, H_2_O_2_ or H_2_O_2_ and CeO_2_NPs. In contrast, cells treated with H_2_O_2_ presented a higher content of 7β-hydroxycholesterol. Treatment with CeO_2_NPs reduced the concentration of oxysterols, although this decrease was not statistically significant (Figure 3B). Similar results were obtained when the ratio of oxysterol/cholesterol was calculated.

### 3.6. Effects of CeO_2_NPs in Total FA Content of HepG2 Cells Treated with Oleic and Palmitic Acid

**Figure 4:**
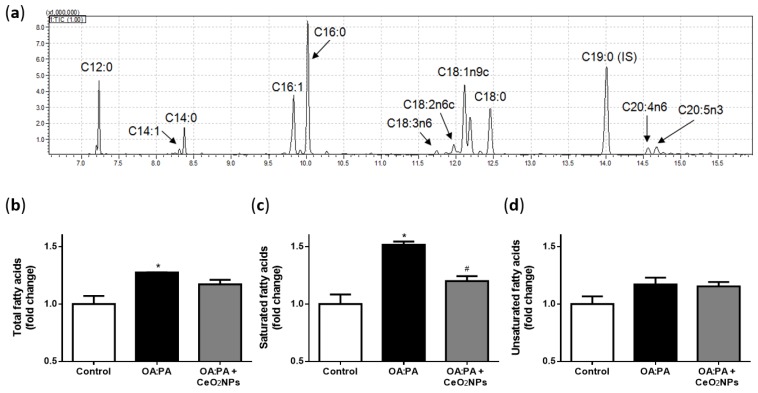
(**a**) Gas chromatography–mass spectrometry chromatogram of fatty acids in HepG2 cells. Content of total fatty acids (**b**), saturated fatty acids (**c**) and unsaturated fatty acids (**d**) in HepG2 cells exposed to vehicle (control), oleic acid:palmitic acid (OA:PA) (1.33:0.67 mM) and OA:PA with CeO_2_NPs (10 µg/mL). * *p* < 0.05 compared with control; # *p* < 0.05 compared with OA:PA.

Oleic and palmitic acid treatment of HepG2 was used as an in vitro model to investigate the effect of CeO_2_NPs in steatosis. HepG2 cells were exposed to 1.33 mM oleic acid (OA) and 0.67 mM palmitic acid (PA) for 24 h and intracellular accumulation of total FAs was measured by GC–MS. A chromatogram showing FAs in HepG2 cells is presented in Figure 4A. Consistent with the induction of the steatosis, total FAs content in HepG2 cells was increased in cells exposed to OA and PA in comparison to those exposed to vehicle (Figure 4B). Treatment with CeO_2_NPs of cells exposed to OA and PA produced a significant reduction of total saturated FAs (Figure 4C), while the total amount of unsaturated FAs was not significantly reduced (Figure 4D).

### 3.7. CeO_2_NPs Induce Specific Changes in the FA Metabolism of Steatotic HepG2 Cells

**Figure 5:**
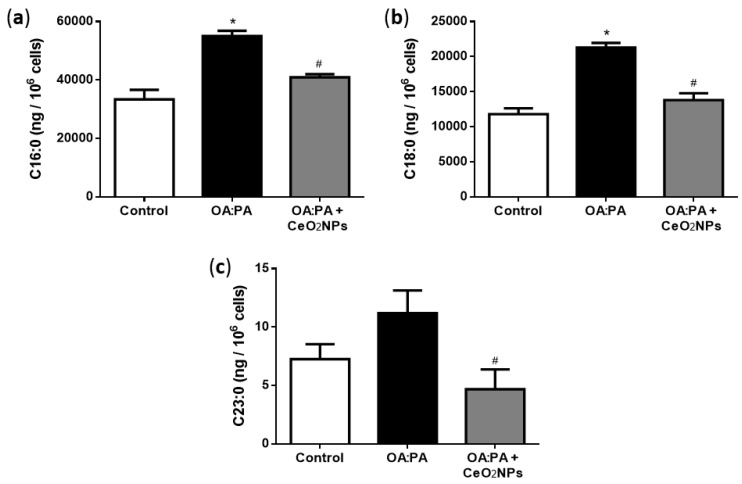
Palmitic (C16:0) (**a**), stearic (C18:0) (**b**) and tricosanoic (C23:0) (**c**) acid content in control cells, cells incubated with OA:PA (1.33:0.67 mM) and cells incubated with OA:PA treated with CeO_2_NPs (10 µg/mL). * *p* < 0.05 compared with control; # *p* ≤ 0.05 compared with OA:PA.

**Figure 6:**
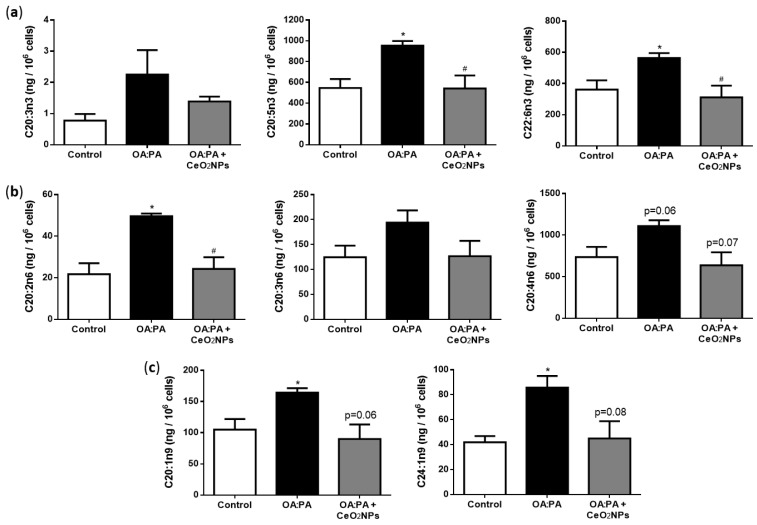
Cell content of n-3 (**a**), n-6 (**b**) and n-9 (**c**) unsaturated fatty acids larger than 18 carbons in control cells, cells incubated with OA:PA (1.33:0.67 mM) and cells incubated with OA:PA treated with CeO_2_NPs (10 µg/mL). * *p* < 0.05 compared with control; # *p* ≤ 0.05 compared with OA:PA.

Analysis of the individual FA species showed that, compared to normal cells, HepG2 cells exposed to OA and PA presented significant elevations in the intracellular content of different FA. As represented in Figure S2 and Table S1 (Supplementary Material), OA and PA incubation increased the cell content of C16:0, C18:0, C18:1n9c, C20:0, C20:1n9, C20:2n6, C20:5n3, C22:6n3 and C24:1n9. Interestingly, CeO_2_NPs treatment of cells exposed to OA and PA decreased the cell content of most of these FA. Thus, CeO_2_NPs treatment resulted in a 26% and 35% significant reduction of the highly abundant saturated FAs C16:0 and C18:0, respectively, and also, significantly decreased the content of very-long-chain saturated FA C23:0 by 58% (Figure 5). Regarding unsaturated FA, CeO_2_NPs reduced the content of most FAs consisting of more than 18 carbon atoms, such as C20:1n9, C20:2n6, C20:3n6, C20:4n6, C20:5n3, C22:6n3 and C24:1n9 (Figure 6). In contrast, C18:3n3 and C18:3n6 content increased 44% and 10%, respectively, in cells treated with CeO_2_NPs. 

**Figure 7:**
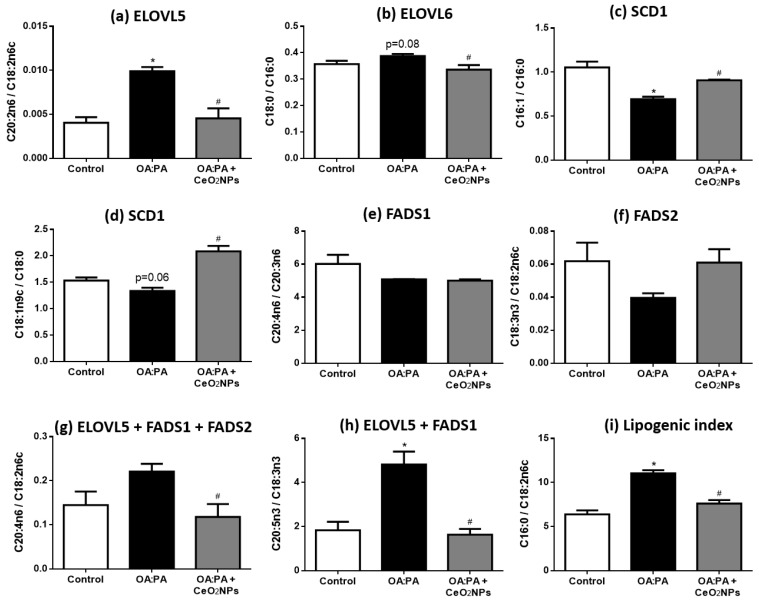
Fatty acid elongase and desaturase activities and lipogenic index in control cells, cells incubated with OA:PA (1.33:0.67 mM) and cells incubated with OA:PA treated with CeO_2_NPs (10 µg/mL). C20:2n6/C18:2n6c (ELOVL5) (**a**); C18:0/C16:0 (ELOVL6) (**b**); C16:1/C16:0 (SCD1-C16:0) (**c**); C18:1n9c/C18:0 (SCD1-C18:0) (**d**); C20:4n6/C20:3n6 (FADS1) (**e**); C18:3n3/C18:2n6c (FADS2) (**f**); C20:4n6/C18:2n6c (ELOVL5 + FADS1 + FADS2 n-6 pathway) (**g**); C20:5n3/C18:3n3 (ELOVL5 + FADS1 n-3 pathway) (**h**); C16:0/C18:2n6c (lipogenic index) (**i**). * *p* < 0.05 compared with control; # *p* < 0.05 compared with OA:PA.

**Scheme 1:**
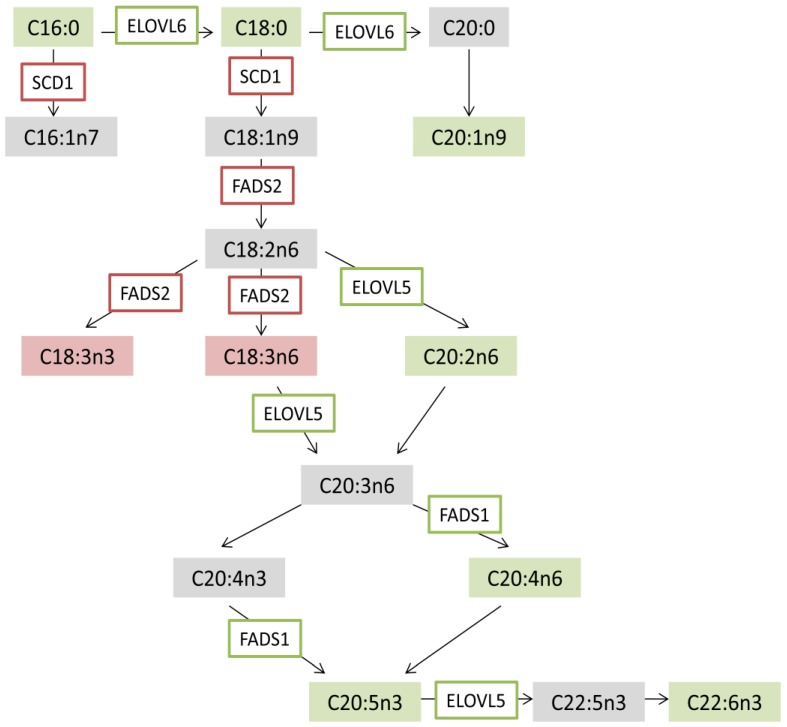
Changes in fatty acid metabolism induced by CeO_2_NPs in steatotic hepatic cells (HepG2 cells). Fatty acids in red are those up (increased content) and in green those down (decreased content) after CeO_2_NPs treatment in our analyses. An increase in elongase and desaturase activities is framed in red and a decrease, in green.

In order to determine the mechanism of CeO_2_NPs antisteatotic effect, metabolic pathways of FAs were investigated. The activities of the main FA elongases (elongation of very-long-chain fatty acids 5 and 6 (ELOVL5 and ELOVL6)), and desaturases (steroyl-CoA desaturase 1 (SCD1) and fatty acid desaturase 1 and 2 (FADS1 and FADS2)), were estimated by measuring the ratios between their products and substrates (Figure 7). Results showed that steatotic HepG2 cells presented an elevation of the lipogenic index C16:0/C18:2n6, which was associated with an activation of the elongase ELOVL5 and an inhibition of the desaturase SCD1 activities. CeO_2_NPs treatment reduced the lipogenic index and reversed the changes in the elongase and desaturase activities induced by OA and PA. In addition, steatosis-induced elongation and desaturation towards the n-3 and n-6 pathway were normalized after steatotic cells were treated with CeO_2_NPs. Scheme 1 shows specific changes in FA metabolism induced by CeO_2_NPs in steatotic cells.

## 4. Discussion

The current study shows that CeO_2_NPs present protective cellular effects and reduce FA content in cultured human hepatic cells (HepG2 cells) cultivated under characteristic conditions of NAFLD. Specifically, cells were cultured under oxidative stress (H_2_O_2_ induced) or steatosis (OA:PA induced) in order to evaluate if CeO_2_NPs presented a direct effect in these specific conditions. First, we evaluated whether CeO_2_NPs were uptaken by HepG2 cells since, after intravenous administration of CeO_2_NPs (3–20 nm), they have been observed to be located within hepatocytes of healthy mice [37] and rats with advanced liver disease [27]. In agreement with these in vivo studies, TEM images obtained in the current study revealed the uptake by HepG2 cells of CeO_2_NPs, which were confined in vacuoles or endosome-like organelles. Similar observations have been reported when studying the uptake of CeO_2_NPs by human hepatic cells C3A, in which confocal microscopy revealed a main perinuclear location of nanoparticles that suggested their presence in late endocytic or secretory compartments [38]. Furthermore, internalization of CeO_2_NPs has been detected in human hepatic cells WRL-68 by flow cytometry [39].

Increased hepatic oxidative stress is a main physiopathological characteristic of NAFLD [40,41]. Increased ROS generation triggers lipid peroxidation, release of inflammatory cytokines and cell death. Patients with NASH display both an increase in ROS and nitrogen species production and a lack of endogenous antioxidant defenses [40]. In this regard, we next evaluated whether CeO_2_NPs could directly decrease cellular oxidative stress in hepatic cells cultured with hydrogen peroxide (H_2_O_2_). CeO_2_NPs produced a reduction in H_2_O_2_-induced cellular oxidative stress, as indicated by the decreased fluorescence of the oxidant-sensitive dye 2′,7′-DCF-DA. This reduction in oxidative stress in CeO_2_NPs-treated cells was accompanied by an improvement in cellular viability measured by the MTS assay. These results indicate that CeO_2_NPs directly protect human hepatic cells from oxidative stress and the associated cellular injury. In agreement with this observation, Azari et al. [42] recently found that pretreatment of HepG2 cells with CeO_2_NPs prevented oxidative and cellular damage caused by acrylamide, a toxic chemical compound present in cooked foods.

Also related to enhanced ROS production in NAFLD is the increase of hepatic oxidized cholesterol metabolites, which have been related to the progression of NAFLD [6,7]. Free cholesterol is susceptible to enzymatic and non-enzymatic oxidation, leading to the formation of a number of cholesterol oxidation products, including 25-hydroxycholesterol (enzymatic reaction) and 7β-hydroxycholesterol and 7-ketocholesterol (non-enzymatic) [43]. Our findings indicate an increase in 7β-hydroxycholesterol, 7-ketocholesterol and 25-hydroxycholesterol when HepG2 cells were exposed to an oxidant stimulus such as H_2_O_2_, suggesting that this is a suitable in vitro model for the study of cholesterol oxidation. However, when cells were co-incubated with CeO_2_NPs, no significant reduction of oxysterols levels was observed. Correction of oxysterols levels by cholesterol lead to a similar trend. Further studies are necessary to ascertain the effect of CeO_2_NPs on oxysterol formation in liver cells.

NAFLD is usually characterized by the presence of hepatic liver steatosis [9,10]. There is evidence derived from animal in vivo studies that CeO_2_NPs treatment may reduce liver steatosis in different models of liver disease. Thus, in previous studies, our group first observed a reduction in liver steatosis in a CCl_4_-induced model of liver fibrosis [27], and the same effect was reproduced in a methionine- and choline-deficient diet model of steatohepatitis in rats [44]. Also, oral administration of CeO_2_NPs to a NAFLD rat model associated with neonatal monosodium-glutamate-induced obesity has been reported to decrease body weight and liver steatosis [28]. However, it is unknown whether these antisteatotic effects are only related to a systemic and/or hemodynamic improvement, or if CeO_2_NPs may also behave as antisteatotic agents per se. In addition, it is unknown whether similar antisteatotic effects of CeO_2_NPs to those observed in rats may be found in the human liver. Interestingly, there is evidence that CeO_2_NPs may interfere in the normal FA metabolism of human-derived hepatocytes (HepG2 cells) [45,46], which represent the cell type in the liver that mainly accumulates FAs in NAFLD.

Therefore, in order to evaluate the ability of CeO_2_NPs to reduce FA content in human hepatic cells, we next used an in vitro model of hepatocellular steatosis based on the administration of OA and PA (2:1 molar ratio) to HepG2 cells. Induction of steatosis in hepatic cells through incubation with a mixture of OA (C18:1n9) and PA (C16:0) is an in vitro steatotic model widely used that is useful to evaluate antisteatotic effects in hepatic cells since it mimics the liver steatosis of NAFLD in patients [30,31,47,48]. OA, a monounsaturated FA, and PA, a saturated FA, are the most abundant FAs present in liver triglycerides [49] and serum [50]. These FAs have individual cell effects: OA is more steatogenic, while PA has a greater pro-apoptotic effect [31]. Exposure of HepG2 cells to a 2 mM mixture of OA:PA with a ratio of 2:1 for 24 h provides the maximal lipid accumulation with minimal cytotoxicity [30,31]. Gómez-Lechón et al. [30] found similar lipid accumulation in this in vitro model of hepatocellular steatosis when compared to hepatocytes from a human steatotic liver.

Our data show that HepG2 cells incubated with OA and PA increased C16:0, C18:0, C18:1n9c, C20:0, C20:1n9, C20:2n6, C20:5n3, C22:6n3 and C24:1n9. This is in agreement with a general induction of FA content previously observed in this model [31] and in the liver of patients with NAFLD [32,51,52,53]. However, C18:3n3 and C18:3n6 levels were not increased. In agreement with this, other research groups found low hepatic levels of C18:3n3 and C18:3n6 in animal models of NAFLD induced by a high-fat diet and CCl_4_ [54] and a methionine- and choline-deficient diet [55], respectively.

PA (C16:0) increases the synthesis of C18:0 by elongation (ELOVL6), whereas OA (C18:1n9c) leads to higher content of C20:1n9 and C24:1n9, according to the metabolic elongation pathway of n-9 fatty acids. OA also promotes the formation of n-3 and n-6 long chain FAs through FADS2. Steatotic HepG2 cells presented decreased contents of C18:3n3 and C18:3n6, which may be associated with reduced FADS2 activity. By contrast, C20:4n6, C20:5n3 and C22:6n3 were markedly increased, supporting an induction of elongation and desaturation towards the n-3 and n-6 pathways. Overall, the increase in the lipogenic index C16:0 / C18:2n6c on HepG2 cells exposed to OA and PA is also in agreement with the induction of the steatotic metabolic process. Other indexes, such as C18:0/C16:0 (ELOVL6), which is increased in this model, have been shown to relate to the degree of liver steatosis in patients with NAFLD [51].

The antisteatotic effect of CeO_2_NPs was proven by reduction of the highly abundant saturated FA C16:0 and C18:0 levels, together with a reduced content of most unsaturated FAs consisting of more than 18 carbon atoms. A differential effect of CeO_2_NPs on C16:0 and C18:1n9 may be explained by the fact that C16:0 is not readily esterified and, to avoid toxicity, is converted to unsaturated FAs (such as C16:1n7 and C18:1n9) through elongation by EVOLV6 and desaturation by SCD1. Our data show that CeO_2_NPs decreased ELOVL6 and increased SCD1 activities, which may explain why C16:1n7 and C18:1n9 levels were not reduced after CeO_2_NPs treatment. Results suggest that CeO_2_NPs may induce a more effective transformation of C16:0 to these unsaturated FAs to avoid toxicity.

The other significant FAs for which contents were reduced by CeO_2_NPs treatment were those unsaturated FAs that consisted of more than 18 carbon atoms. The effect of CeO_2_NPs in the reduction of these FAs was remarkable since many of them were completely normalized. This effect may be related to the observed inhibition of elongation and desaturation towards the n-3 and n-6 pathway. Importantly, CeO_2_NPs reduced the eicosanoid precursors C20:4n6, C20:5n3 and C22:6n3, which are involved in the induction of inflammation and cell membrane integrity. However, provided that FAs consisting of more than 18 carbon atoms are preferentially oxidized in peroxisomes rather than in mitochondria, further research is necessary to evaluate whether CeO_2_NPs may also promote peroxisome FA oxidation. In addition, C18:3n6 and C18:3n3 FA, which were not increased in steatotic HepG2 cells, exhibited an increase in their levels when cells were treated with CeO_2_NPs, suggesting a CeO_2_NPs-mediated induction of FADS2 activity. Finally, reduction of the lipogenic index by CeO_2_NPs supports an overall antisteatotic effect.

One limitation of this study relates to the lack of evaluation of other factors that may also play a role in the pathophysiology of NAFLD, together with oxidative stress and lipid dysregulations. A significant example are the nuclear receptors, such as peroxisome proliferator-activated receptors (PPARs), liver X receptors (LXR) and farnesoid X receptors (FXR). They have been involved in the natural history of NAFLD, demonstrating that PPARs have one of the main roles in hepatic steatogenesis, LXR in hepatic inflammation and FXR in hepatic fibrogenesis [56]. Furthermore, TEM images showing the cell uptake of CeO_2_NPs were only obtained in basal conditions and there could be some changes in the localization of CeO_2_NPs under oxidative stress and/or steatotic conditions. Finally, the antisteatotic effect of CeO_2_NPs was evaluated in an OA:PA mixture and differential effects on steatosis induced by either OA alone or PA alone were not evaluated.

## 5. Conclusions

CeO_2_NPs directly protect human hepatic cells from cell injury in conditions of oxidative stress and reduce FA content in steatotic conditions by inducing specific metabolic changes, suggesting that these NPs have great potential in the treatment of NAFLD.

## Supplementary Materials

The following are available online at https://www.mdpi.com/2218-273X/9/9/425/s1.
