# Supplementary material for: Beyond the Scavenging of Reactive Oxygen Species (ROS): Direct Effect of Cerium Oxide Nanoparticles in Reducing Fatty Acids Content in an In Vitro Model of Hepatocellular Steatosis
Source: Biomolecules. 2019 Aug 29;9(9):425. doi: 10.3390/biom9090425 (PMC6770635; doi:10.3390/biom9090425)
Supplement: Supplementary file 1 [file biomolecules-09-00425-s001.pdf]

Figure S1. Cell viability (**a**) and intracellular reactive oxygen species (ROS) production (**b**) of HepG2 cells incubated with medium (control), H_2O_2 (1.5 mM) and H_2O_2 and CeO_2NPs (1.5 mM; 10 µg/mL). *p < 0.05 vs control and #p < 0.05 vs H_2O_2. **Figure S2**: Content of saturated (**a**), monounsaturated (**b**) and polyunsaturated (**c**) fatty acids in HepG2 cells exposed to vehicle (control), OA:PA (1.33:0.67 mM) and OA:PA with CeO_2NPs (10 µg/mL). **Table S1**: Fatty acids content in HepG2 cells exposed to vehicle (control), OA:PA (1.33:0.67 mM) and OA:PA with CeO_2NPs (10 µg/mL).

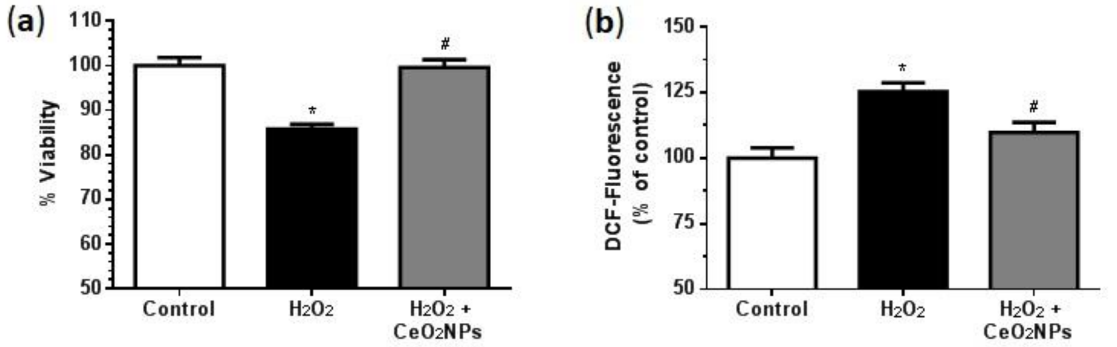

Figure S1. Cell viability (**a**) and intracellular ROS production (**b**) of HepG2 cells incubated with medium (control), H_2O_2 (1.5 mM) and H_2O_2 and CeO_2NPs (1.5 mM; 10 µg/mL). *p < 0.05 vs control and #p < 0.05 vs H_2O_2.

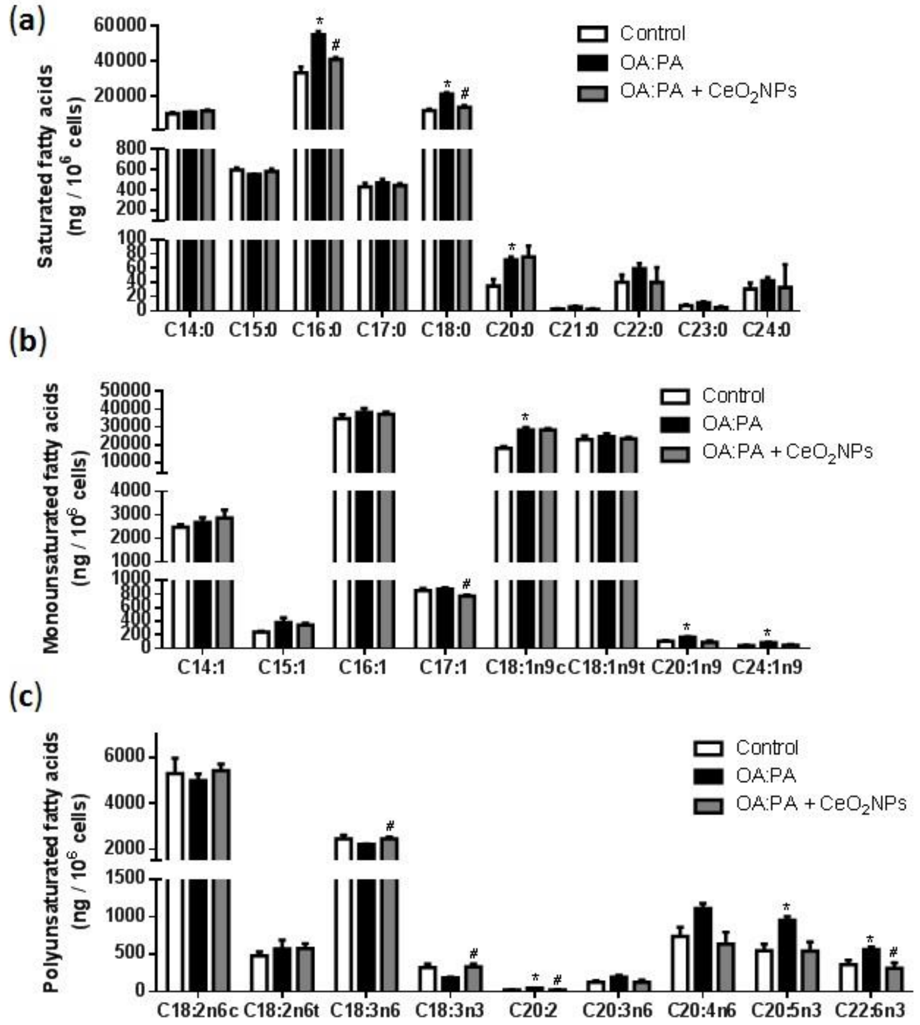

Figure S2. Content of saturated (**a**), monounsaturated (**b**) and polyunsaturated (**c**) fatty acids in HepG2 cells exposed to vehicle (control), OA:PA (1.33:0.67 mM) and OA:PA with CeO_2NPs (10 μg/mL). *$p < 0.05$ compared with control; #$p < 0.05$ compared with OA:PA.

Table S1. Fatty acids content in HepG2 cells exposed to vehicle (control), OA:PA (1.33:0.67 mM) and OA:PA with CeO_2NPs (10 μg/mL).

Fatty acids (ng / 10^6 cells)	Control	OA:PA	OA:PA+ CeO_2NPs
C12:0	120.0 ± 62.10	210.1 ± 14.52	291.8 ± 104.7
C14:0	10168 ± 661.7	11075 ± 102.1	11769 ± 376.6
C14:1	2490 ± 103.9	2673 ± 221.5	2864 ± 350.7
C15:0	594.8 ± 21.84	552.1 ± 4.895	580.6 ± 25.00
C15:1	242.5 ± 7.916	380.2 ± 72.78	345.6 ± 28.20
C16:0	33369 ± 3277	55013 ± 1837*	40922 ± 1069#
C16:1	34617 ± 2253	38144 ± 2298	37115 ± 1220
C17:0	429.5 ± 35.39	469.9 ± 35.45	442.0 ± 19.78
C17:1	845.6 ± 37.01	873.5 ± 16.63	767.3 ± 19.01#
C18:0	11793 ± 840.9	21290 ± 657.8*	13797 ± 984.3#
C18:1n9c	17980 ± 1108	28358 ± 1335*	28373 ± 747.6
C18:1n9t	22926 ± 1953	24671 ± 1517	23429 ± 616.6
C18:2n6c	5308 ± 661.7	5006 ± 280.0	5429 ± 284.2
C18:2n6t	480.0 ± 49.83	571.0 ± 115.5	577.6 ± 65.52
C18:3n6	2452 ± 162.6	2216 ± 11.28	2462 ± 72.71#
C18:3n3	319.4 ± 50.17	186.7 ± 10.35	330.5 ± 42.42#
C20:0	34.99 ± 9.240	71.35 ± 4.289*	75.35 ± 15.60
C20:1n9	104.9 ± 16.99	164.3 ± 7.179*	89.98 ± 23.33
C20:2n6	21.74 ± 5.267	49.63 ± 1.268*	24.29 ± 5.560#
C20:3n6	124.6 ± 23.29	194.0 ± 24.51	126.7 ± 30.63
C20:3n3	0.775 ± 0.2146	2.250 ± 0.7858	1.388 ± 0.1573
C20:4n6	735.2 ± 122.6	1108 ± 69.95	636.8 ± 156.1
C20:5n3	546.6 ± 86.09	953.4 ± 45.37*	541.5 ± 125.0#
C21:0	2.475 ± 0.725	5.850 ± 1.126	2.200 ± 0.950
C22:0	40.01 ± 10.02	59.25 ± 7.039	39.84 ± 20.82
C22:2	9.463 ± 3.798	13.67 ± 1.349	10.11 ± 2.993
C22:6n3	361.1 ± 59.02	563.4 ± 31.29*	311.1 ± 75.12#
C23:0	7.250 ± 1.286	11.20 ± 1.935	4.680 ± 1.696#
C24:0	30.80 ± 8.530	42.28 ± 4.217	32.93 ± 32.03
C24:1n9	41.86 ± 5.048	85.72 ± 9.331*	44.92 ± 13.88
C22+23+24	78.06 ± 19.68	112.7 ± 13.11	61.78 ± 36.45
Total FA	146135 ± 10387	186338 ± 115.4*	171210 ± 5788
Saturated FA	56589 ± 4713	85793 ± 1609*	67884 ± 2461#
Unsaturated FA	89547 ± 6021	104830 ± 5350	103327 ± 3364

Mean ± SEM. *$p < 0.05$ compared with control; #$p < 0.05$ compared with OA:PA.
